# Uchimata: a toolkit for visualization of 3D genome structures on the web and in computational notebooks

**Published:** 2025-09-16

**Authors:** David Kouřil, Trevor Manz, Tereza Clarence, Nils Gehlenborg

**Affiliations:** 1 Harvard Medical School, Boston, MA, USA; 2 Icahn School of Medicine at Mount Sinai, New York, NY, USA

## Abstract

**Summary:**

Uchimata is a toolkit for visualization of 3D structures of genomes. It consists of two packages: a Javascript library facilitating the rendering of 3D models of genomes, and a Python widget for visualization in Jupyter Notebooks. Main features include an expressive way to specify visual encodings, and filtering of 3D genome structures based on genomic semantics and spatial aspects. Uchimata is designed to be highly integratable with biological tooling available in Python.

**Availability and Implementation:**

Uchimata is released under the MIT License. The Javascript library is available on NPM, while the widget is available as a Python package hosted on PyPI. The source code for both is available publicly on Github (https://github.com/hms-dbmi/uchimata and https://github.com/hms-dbmi/uchimata-py). The documentation with examples is hosted at https://hms-dbmi.github.io/uchimata/

**Contact:**

david_kouril@hms.harvard.edu or nils@hms.harvard.edu.

## Introduction

Alongside sequencing-based technologies developed to probe the spatial conformation of genomes—such as the influential Hi-C method (Lieberman-Aiden et al. 2009)—there are ongoing efforts to produce concrete 3D models that depict genome folding. Although research on macromolecular structures has long benefited from sustained investment in resources such as the Protein Data Bank (Berman et al. 2000) and visualization tools like PyMOL (Schrödinger, LLC 2015) and Mol* (Sehnal et al. 2021), comparable tools for analysis and visualization of physical genome structures remain limited.

There are two principal approaches to generating structural models of genomes (Imakaev, Fudenberg, and Mirny 2015): *data-driven* methods, which produce structures that satisfy constraints derived from input data (e.g., Hi-C), and methods that simulate structures *de novo* based on mechanistic principles. Notable examples of existing structures include Duan et al.’s yeast genome model (Duan et al. 2010), Stevens et al.’s mammalian (mouse) genome structures constructed from single-cell Hi-C (Stevens et al. 2017), and Tan et al.’s genome structures of single diploid human cells (Tan et al. 2018). Recent studies show promise in employing machine learning techniques to infer 3D genome structures (Schuette, Lao, and Zhang 2025). Beyond individual publications, there have been efforts to develop centralized databases of 3D genome models (Oluwadare et al. 2020), although community adoption remains limited.

Visualizing specialized three-dimensional data can be challenging for computational biologists lacking computer graphics expertise, who depend on tools that abstract low-level rendering details. Although several tools have been developed for *visualizing 3D genome* structural data—such as Genome3D (Asbury et al. 2010), 3DGB (Butyaev et al. 2015), GMOL (Nowotny et al. 2016), HiC-3DViewer (Djekidel et al. 2017), and CSynth (Todd et al. 2021)—most exist as standalone desktop or web applications, making them difficult to integrate into notebook-based analytical workflows, to adapt for case-specific applications, and to customize beyond the visual choices predefined by their developers.

Two genome browsers—WashU Epigenome (Li et al. 2022) and Nucleome Browser (Zhu et al. 2022)—currently provide visualization capabilities for 3D genome models. These remain the most comprehensive toolkits available, integrating diverse genomics data types and visualizations. However, their integrative power still falls short compared to computational notebooks, which enable flexible combination of software packages through end-user programming (Ko et al. 2011) to support novel analytical scenarios. Computational notebooks have become the de facto standard for analysis in computational biology and are commonly used to interface with simulation packages that generate 3D genome structures. Despite this, visualization support for such data within notebook environments has lagged behind.

To address the lack of dedicated visualization tools in computational notebooks, scientists often repurpose general plotting libraries or tools originally designed for protein structures. For instance, nglutils (https://github.com/mirnylab/nglutils) builds on the molecular viewer nglview (Nguyen, Case, and Rose 2018) to support interactive visualization of genome structures within notebooks. Similarly, some simulation packages for 3D genome structures recommend using general visualization libraries, such as matplotlib (Hunter 2007) or fresnel (https://github.com/glotzerlab/fresnel).

**Figure 1.**
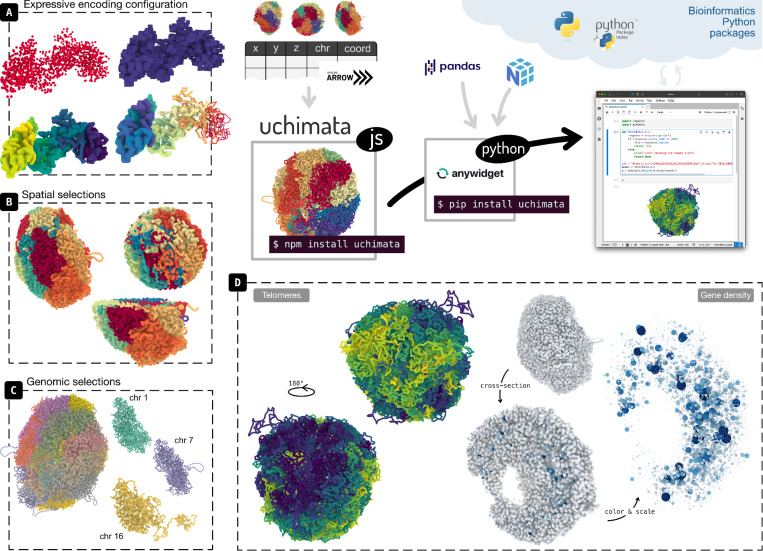
Uchimata consists of two packages, Javascript and Python, intended to cover use cases for visualization of three-dimensional genome models across the web and computational notebooks. Key features include expressive configuration of visual encoding (A), selections based on spatial attributes (B), and selections based on genomic coordinates (C). Two use cases illustrating situations where 3D visualization brings new insights (D): mapping bin coordinates to continuous color scale shows concentration of telomeres on one side of the structures (left), and encoding gene density to color and scale of the bin marks shows concentration of gene-rich regions within the structures, further from the boundary (right).

We developed **uchimata**, a toolkit for visualizing 3D genome structures across the web and computational notebook environments (Fig. 1). Uchimata aims to provide a high-level application programming interface (API) with genomics context. Furthermore, we emphasize alignment with existing bioinformatics tools and focus on making uchimata composable in several usage scenarios. Finally, uchimata adopts a grammar-of-graphics approach (Wilkinson 2005) in order to allow a more expressive definition of the visualization design.

## Methods

### Design of the uchimata toolkit

The design of uchimata is guided by the principle of composability (Manz 2024). To support long-term maintainability and enable the reuse of visualization tools as components in novel scenarios, each tool should focus on a narrow and well-defined set of features. In uchimata, our focus is on visualizing 3D models, while related representations are deliberately left to other specialized tools. This modular approach allows users to select and combine components to construct interfaces and pipelines tailored to their specific requirements, avoiding the pitfalls of monolithic software that becomes progressively difficult to extend and maintain.

The second guiding principle is the **separation of concerns** and leveraging strengths of individual programming environments. Over time, the Javascript and Python ecosystems have developed distinct competencies. The web platform excels at building user interfaces and offers a wide range of visualization libraries. Its core technologies—HTML, CSS, and Javascript—benefit from standardization and strong backward compatibility. Python, by contrast, is well suited for data wrangling and analysis, and has become a dominant platform in computational biology. Some libraries, such as NumPy (Harris et al. 2020) and pandas (McKinney 2010), introduced concepts now considered core to the Python ecosystem, even though they exist outside the language and its standard library.

Finally, uchimata embraces existing **data standards**, some of which reach beyond genomics or bioinformatics. This approach allows it to benefit from mature infrastructure developed by broader communities. For example, uchimata uses Apache Arrow (https://arrow.apache.org) as an in-memory representation of 3D genome structures, avoiding the need to implement custom loaders for the diverse formats in which these structures are typically stored—such as the PDB file format. In computational notebooks, uchimata accepts input data as pandas DataFrames and NumPy arrays, embracing canonical data structures in scientific Python. The prevalence of these formats increases the likelihood of future integrations through these interfaces and compatibility with other toolkits.

### Implementation

The core web uchimata library (https://github.com/hms-dbmi/uchimata) is implemented using Typescript, transformed into a standard Javascript module, and hosted on NPM. The Jupyter notebook widget (https://github.com/hms-dbmi/uchimata-py) is built using anywidget (Manz, Abdennur, and Gehlenborg 2024) and uses the Javascript library to serve the canvas within a notebook’s cell. The 3D graphics rendering is accomplished through three.js, currently using the WebGL backend. We use DuckDB (Raasveldt and Mühleisen 2019) to perform a variety of queries to filter the Apache Arrow tables representing the 3D structures and associated data.

## Results

The development of uchimata was driven by the aim to support a broad range of end-use scenarios. We focused on core functionality that could be reused in web-based applications with case-specific interfaces, without overloading the core library with features relevant only to a narrow subset of users. We also envisioned seamless integration of uchimata into data portals.

A second major group of use cases involves visualization in exploratory stages, such as during simulations and in downstream analyses of simulated data. While the library can be used directly in Javascript-based notebook environments such as Observable Notebooks (https://observablehq.com/platform/notebooks), much computational notebook work today is carried out in Python, leveraging its extensive scientific software ecosystem.

### Integration in web applications & Javascript-based notebooks

In the first scenario, uchimata functions like any other Javascript library in a web environment, enabling visualization of 3D genome structural data. To show uchimata’s ability to support novel visualizations, we use it within Observable Notebooks—a literate programming environment, similar to Jupyter Notebooks, that executes Javascript code in interactive cells. A set of examples is available at https://hms-dbmi.github.io/uchimata/, source code for these examples is in the uchimata Github repository, under the `docs` folder. At its core, a 3D genome structure is a list of XYZ coordinates, sometimes associated with genomic coordinates. How these data items are visually represented is left to the user, who specifies a *view config* that maps items to concrete *marks*, such as spheres or cubes, and their visual features such as *color* and *scale*. A *scene* contains an array of structures, each paired with its corresponding view config, which together define how the structures are rendered.

We envision uchimata being integrated into use case-specific web applications and genome browsers. We recently used uchimata to extend the Gosling grammar (L’Yi et al. 2022) with support for 3D genome models (Kouřil et al. 2025), thereby implementing the previously recognized ‘spatial’ layout for genomic coordinate systems (Nusrat, Harbig, and Gehlenborg 2019) that was missing in existing grammar-based tools. This grammar-of-graphics approach allows for both expressiveness and reproducibility, and by unifying 3D representations of genomes with conventional genomic data views, it opens new avenues for exploring efficient visual linking between distant genomic loci.

### Interoperability with existing bioinformatics workflows in Python

Computational notebooks play an important role in day-to-day analytical work of genomics researchers (van den Brandt et al. 2025). In the second scenario, uchimata can be used as a widget for Python-based computational notebooks.

Python is often used for chromatin simulations, where visual inspection is typically the first step in assessing the results. To shorten the iteration loop, a visualization tool should be available directly within the simulation environment, enabling biologists to perform basic checks, adjust parameters, and rerun simulations. The uchimata widget offers multiple ways to input data which aligns with in-memory storage practices in Python-based computation notebooks. Most relevant for Jupyter environments are widely used data structures such as NumPy arrays and pandas DataFrame.

Furthermore, the availability through Python allows for integration with existing bioinformatics tools. For example, we can combine uchimata with bioframe (Open2C et al. 2024) to apply genomic range selections on 3D genome structures. Bioframe offers functionality for loading genomic data from typical formats such as BED, GFF, or GTF, and performing a variety of interval operations, representing the ranges as pandas DataFrames. Uchimata then accepts these dataframes as selection queries and outputs corresponding bins of the 3D structure. The user can thus apply the same range as selection across multiple different 3D structures, facilitating comparison across an ensemble of simulated structures. In addition to genomics-based filtering, uchimata supports spatial selections (Fig. 1B), currently implementing cutting-plane (cross-section) operations and selecting spherical neighborhoods of a specified radius.

Fig. 1D highlights two examples that demonstrate how visualization of 3D models reveals insights about genome structure. First, we use Stevens et al.’s mouse cell structures (Stevens et al. 2017) and encode each chromosome’s coordinates with a continuous color scale. This results in clear identification of locations where telomeres concentrate (Fig. 1D, left). Second, we aggregate gene annotation loaded from a GTF into bins matching the resolution of the structure. We then map this gene density data to both color and scale of the marks representing the bins (Fig. 1D, right). Scaling the marks can act as a form of removing occlusion and highlighting inner structure. The biological insight in this example is that gene-rich regions concentrate in the center of the genome structure, further from the border.

## Conclusion

While the heatmap representation (i.e., the contact matrices resulting from Hi-C) remains the most direct experimental way to observe how whole genomes fold in nuclear space, we believe that the three-dimensional structural representation offers a complementary benefit. Its primary strength is that it contextualizes genomic data within 3D space, which can lead to hypotheses related to specific patterns observed in the actual 3D structure. With uchimata, we contribute software that makes it easier to visualize this type of genomics data. With this crucial infrastructure in place, we intend to further investigate means of linking between traditional genome browser views, dense multiscale matrix viewers, and three-dimensional structure visualizations. On the rendering side, we plan to adopt the WebGPU API as it becomes broadly supported across major web browsers.
